# Assessment of Fatigue and Associated Factors in Patients with Inflammatory Bowel Disease: A Questionnaire-Based Study

**DOI:** 10.3390/jcm12093116

**Published:** 2023-04-25

**Authors:** Han Hee Lee, Tae-Geun Gweon, Sung-Goo Kang, Sung Hoon Jung, Kang-Moon Lee, Sang-Bum Kang

**Affiliations:** 1 Department of Internal Medicine, College of Medicine, The Catholic University of Korea, Seoul 06591, Republic of Korea; hanyee99@hanmail.net (H.H.L.); gweontae@naver.com (T.-G.G.); drmaloman@catholic.ac.kr (K.-M.L.); 2 Department of Family Medicine, St. Vincent’s Hospital, College of Medicine, The Catholic University of Korea, Seoul 16247, Republic of Korea

**Keywords:** inflammatory bowel disease, fatigue, questionnaires, disease activity, risk factors

## Abstract

Although fatigue is common in patients with inflammatory bowel disease (IBD), it often goes unrecognized and untreated. We investigated the degree of fatigue and associated factors in patients with IBD. A multicenter study involving 147 IBD patients was conducted at five academic hospitals from August 2019 to December 2021. Fatigue was evaluated using the validated Korean version of the Multidimensional Fatigue Inventory (MFI-K). Among 97 ulcerative colitis patients and 50 Crohn’s disease patients, the mean total MFI-K score was 59.0 ± 5.5, which corresponded to a moderate-to-severe level of fatigue. Moderate-to-severe disease activity was found to be significantly associated with a higher general and physical fatigue subscale MFI-K score compared to remission-to-mild disease activity (17.6 ± 1.7 vs. 16.7 ± 2.0, *p* = 0.009), while the use of biologics was associated with a lower total MFI-K score (57.3 ± 5.0 vs. 59.5 ± 5.5, *p* = 0.031). In multiple linear regression, the total MFI-K score was positively correlated with a history of surgery for IBD, while it was negatively correlated with the use of biologics. Depression was positively correlated with the reduced motivation subscale score. The degree of fatigue in patients with IBD was high. Disease activity, the use of biologics, a history of surgery for IBD, and depression were associated with fatigue.

## 1. Introduction

Inflammatory bowel disease (IBD) is a chronic condition that affects millions of people worldwide, with symptoms that include abdominal pain, diarrhea, and hematochezia. In addition to these physical symptoms, IBD can also have mental and neuropsychiatric manifestations, such as fatigue [1,2]. Fatigue is one of the most frequent symptoms reported by patients with IBD, and its impact can be significant, leading to reduced quality of life, impaired work productivity, and decreased social functioning [3,4].

Fatigue is a complex and multifactorial phenomenon that can involve both mental and physical factors. It refers to a sense of exhaustion or lack of energy that is not in proportion to the level of physical exertion, and which can limit daily activities [5]. Unlike normal tiredness, fatigue may not be relieved by rest. Fatigue has been defined as a persistent overwhelming sense of tiredness, weakness, or exhaustion that can be physical, mental, or both [6]. However, there is still no standardized definition of fatigue and there are no widely accepted diagnostic criteria or measurement tools for assessing fatigue. This has hampered efforts to understand the pathogenesis of fatigue and has made it difficult to develop effective treatments.

The prevalence of fatigue in IBD patients is high, with studies reporting rates of over 80% among patients with active IBD [7,8,9] and 40% among those in remission [9,10,11]. These rates are much higher than those seen in healthy individuals [2,12,13], highlighting the significant impact of fatigue on the bodies and minds of patients with IBD, regardless of the disease’s level of activity. The causes of fatigue in IBD patients are not yet fully understood, but they are believed to be multifactorial, with both physical and psychosocial factors playing a role.

Although patients with IBD may experience fatigue more often than classic symptoms such as urgency or diarrhea, fatigue is often given lower consideration during clinical consultations because of the difficulty in assessing its severity and the lack of established treatment options [14,15,16]. However, fatigue has a significant negative impact on the quality of life for patients with IBD. It can disrupt their daily activities and even lead to loss of employment [17,18,19], resulting in socioeconomic losses. Therefore, identifying the factors associated with fatigue in IBD patients and developing effective treatment strategies are crucial.

Previous studies have investigated the physical factors associated with fatigue in patients with IBD and have reported various significant factors, including disease activity, anemia, medication for IBD such as corticosteroid or immunomodulators, body mass index, and level of physical activity [9,13,18,20,21]. Psychosocial factors, such as anxiety, depression, or sleep disturbance, have also been suggested as associated factors [22,23]. However, the findings across those studies are inconsistent and there remains uncertainty regarding the specific aspects of fatigue that are influenced by such factors.

Therefore, the aims of this study were to investigate the level of fatigue in patients with IBD and to identify the factors that influence fatigue, using a validated Korean questionnaire.

## 2. Materials and Methods

### 2.1. Study Population

This prospective multicenter, cross-sectional study was conducted at five institutions of the Catholic University of Korea (Daejeon St. Mary’s Hospital, Eunpyeong St. Mary’s Hospital, Yeouido St. Mary’s Hospital, Bucheon St. Mary’s Hospital, and St. Vincent’s Hospital) and involved 147 patients diagnosed with ulcerative colitis (UC) or Crohn’s disease (CD) from August 2019 to December 2021. We enrolled patients with IBD who were at least 19 years of age and who were scheduled to undergo laboratory and endoscopic examinations to assess their disease activity. We excluded patients who had difficulty understanding or completing the questionnaires, were pregnant or within 3 months of giving birth. or had comorbidities that cause fatigue. such as heart disease, lung disease, chronic kidney disease, or liver cirrhosis.

### 2.2. Ethical Considerations

This study was conducted in accordance with the Declaration of Helsinki and was approved by the institutional review boards of the participating hospitals (XC19QEDI0052). Written informed consent was obtained from the subjects, who were instructed to respond to all questionnaire items after reading the instructions.

### 2.3. Data

The researchers explained the purpose of the study and the composition and contents of the questionnaire to the enrolled patients. If patients agreed to participate, either the researchers or research nurses provided them with the questionnaire. The patients were given sufficient time to read and respond to the questionnaire items and they could ask the researchers to clarify any ambiguities.

Age, sex, body mass index (BMI), disease duration, and history of surgery for IBD were investigated. Laboratory data pertaining to the patients, i.e., hemoglobin, protein, albumin, the erythrocyte sedimentation rate (ESR), and C-reactive protein (CRP), together with patients’ use of 25-OH-vitamin D and current medications for IBD, were also collected within one month before and after the survey. Anemia was defined as hemoglobin < 13 g/dL for males and <12 g/dL for females [24,25]. Hypoalbuminemia was defined as albumin < 3.5 g/dL. Disease activity was measured using the Mayo score and Crohn’s Disease Activity Index (CDAI) [26,27]. In cases of UC, we defined clinical remission as a Mayo score ≤ 2 and no subscale score > 1, mild activity as a score of 3–5, moderate activity as a score of 6–10, and severe activity as a score of 11–12 [26]. In cases of CD, we defined clinical remission as a CDAI < 150, mild activity as a CDAI ≥ 150 and <220, moderate activity as a CDAI ≥ 220 and <450, and severe activity as a CDAI ≥ 450 [27].

### 2.4. Questionnaires

Fatigue was evaluated using the Korean version of the Multidimensional Fatigue Inventory (MFI). The MFI, developed by E. M. A. Smets in 1995, consists of 20 items divided into five subscales: general fatigue, physical fatigue, reduced activity, reduced motivation, and mental fatigue [28]. The general fatigue subscale measures overall levels of fatigue and exhaustion, while the physical fatigue subscale measures fatigue related to physical activities and exertion. The mental fatigue subscale measures fatigue related to cognitive and mental activities, such as concentration and memory. The reduced activity subscale measures the impact of fatigue on daily activities and functioning, while the reduced motivation subscale measures the impact of fatigue on motivation and drive to engage in activities. Each subscale consists of four items. Each item is rated on a scale of 1–5 according to the individual’s experience. The total MFI score is 20–100 points, and a higher score corresponds to more acute fatigue. The MFI has been validated in a variety of populations, including patients with IBD, cancer, chronic fatigue syndrome, and multiple sclerosis. It has been shown to have good internal consistency, test–retest reliability, and construct validity. The MFI can be used to assess fatigue over time and to identify specific dimensions of fatigue that may be more problematic for individual patients.

Unlike the original version, the Korean version of the MFI (MFI-K) has four subscales; the general and physical fatigue subscales are integrated. The MFI-K has been validated in Korean patients [29].

The Hospital Anxiety and Depression Scale (HADS) was used to assess anxiety and depression [30]. The HADS includes 14 items assessing symptoms of both anxiety (HADS-A subscale) and depression (HADS-D subscale) [31]. Items were scored from 0 to 3, with total scores on each subscale ranging from 0 to 21. A score of ≥8 was taken to indicate the presence of anxiety or depression [30].

### 2.5. Statistical Analysis

Descriptive statistics were generated for the demographic data. Continuous variables were expressed as mean ± standard deviation and were compared by an independent *t*-test or one-way ANOVA, as appropriate. Categorical variables were expressed as numbers (percentage) and were compared between groups using the chi-square test.

Multiple linear regression using backward elimination was conducted to identify factors affecting the MFI-K score. To ensure adequate statistical power for our multiple logistic regression analysis of factors associated with fatigue in patients with IBD, we calculated the required sample size using a sample size calculator. We set the significance level α at 0.05 and the power (1-β) at 0.80. Based on previous studies, we estimated the effect size of odds ratio to be 1.5 and identified three independent variables to include in the model. The estimated prevalence of fatigue in our target population was 50%. Using the sample size calculator, we determined that a minimum sample size of 91 participants would be required to detect significant associations. To address the possibility of dropouts or missing data, we included a 10% margin of error and determined that a minimum sample size of 101 participants was necessary. For the regression analysis, we used age, BMI, and disease duration as continuous variables, while sex, history of surgery for IBD, anemia, hypoalbuminemia, use of steroids, biologics, disease activity (remission to mild vs. moderate to severe), anxiety, and depression were used as dichotomous variables. All analyses were performed using SPSS software (version 21.0; SPSS Inc., Chicago, IL, USA) and a two-sided *p*-value of <0.05 was considered indicative of statistical significance.

## 3. Results

### 3.1. Demographic and Clinical Characteristics

A total of 147 IBD patients, comprising 97 UC and 50 CD patients, were enrolled in this study (Table 1). The mean age of the patients was 36.2 ± 13.5 years and 97 (66.0%) were male. BMI and disease duration were not different between the patients with UC and CD. A history of surgery for IBD was noted in CD patients (*n* = 11, 22%) but not in UC patients (*p* < 0.001). Regarding current medications, UC patients were taking more 5-ASAs and CD patients were taking more immunomodulators, steroids, and biologics. Regarding disease activity, about 40% of the patients were in remission. Mild, moderate, and severe disease activities were exhibited by 27.9%, 27.9%, and 3.4% of the patients, respectively.

The mean total MFI-K score of patients with IBD was 59.0 ± 5.5. The MFI-K, HADS-A, and HADS-D scores did not differ significantly between the UC and CD patients.

### 3.2. Relationship between the MFI-K Score and Various Demographic and Clinical Variables in Patients with IBD

Table 2 shows the total MFI-K scores according to demographic characteristics. Age, sex, BMI, disease duration, anemia, hypoalbuminemia, and use of steroids did not affect the total MFI-K score. Use of biologics significantly decreased the total MFI-K score (57.3 ± 5.0 vs. 59.5 ± 5.5, *p* = 0.031). A history of surgery for IBD and disease activity increased the score, with borderline significance (*p* = 0.085 and *p* = 0.083, respectively).

In the subanalysis of the four MFI-K subscale scores, it was found that higher scores for general and physical fatigue were associated with hypoalbuminemia (18.3 ± 0.5 vs. 16.9 ± 2.0, *p* < 0.001) and moderate-to-severe disease activity (17.6 ± 1.7 vs. 16.7 ± 2.0, *p* = 0.009). Higher scores for reduced activity were associated with male gender (14.1 ± 2.2 vs. 13.2 ± 2.2, *p* = 0.029) and high BMI > 25 kg/m^2^ (14.7 ± 1.9 vs. 13.7 ± 2.2, *p* = 0.029). Higher scores of reduced motivation were associated with both anxiety (12.2 ± 3.9 vs. 9.7 ± 3.1, *p* < 0.001) and depression (12.3 ± 3.4 vs. 9.4 ± 3.3, *p* < 0.001).

### 3.3. Factors Independently Associated with the Total MFI-K Score and MFI-K Subscale Scores

Multiple linear regression was performed to identify factors independently associated with the total MFI-K scores and MFI-K subscale scores (Table 3). A history of surgery for IBD was independently associated with a higher total MFI-K score (regression coefficient, 3.35; 95% confidence interval [CI], 0.01–6.70; *p* = 0.049), while the use of biologics was associated with a lower total MFI-K score (regression coefficient, −2.68; 95% CI, −4.81 to −0.54; *p* = 0.014).

In terms of the MFI-K subscale scores, moderate-to-severe disease activity was independently associated with a higher score for general and physical fatigue compared to remission-to-mild disease activity (regression coefficient, 0.86; 95% CI, 0.15–1.58; *p* = 0.019). Moreover, the use of biologics was associated with a lower score of mental fatigue (regression coefficient, −1.22; 95% CI, −4.81 to −0.54; *p* = 0.014), while anxiety (regression coefficient, −1.31; 95% CI, −2.34 to −0.29; *p* = 0.012) and depression (regression coefficient, −1.34; 95% CI, −2.35 to −0.33; *p* = 0.009) were also associated with a lower score of mental fatigue. Male gender (regression coefficient, −0.99; 95% CI, −1.73 to −0.24; *p* = 0.010) and anxiety (regression coefficient, −1.08; 95% CI, −1.89 to −0.26; *p* = 0.010) were associated with a lower score for reduced activity. Depression was the only independent factor for a higher score for reduced motivation (regression coefficient, 2.86; 95% CI, 1.71–4.01; *p* < 0.001).

## 4. Discussion

In this multicenter prospective study, 147 patients with IBD had high MFI-K scores. The use of biologics was negatively correlated with the total MFI-K score, whereas higher disease activity was associated with increased general and physical fatigue. Depression was positively correlated with the reduced motivation score among the four subscales in the MFI-K. A history of surgery for IBD and the use of biologics were independent predictors of the total MFI-K score.

While the exact mechanisms underlying fatigue are not well understood, it is believed to involve a complex interplay between biological, psychological, and social factors. Chronic inflammation, sleep disturbances, and psychological stress are some of the factors that have been implicated in the development of fatigue in various medical conditions, including IBD [32]. In addition, the subjective nature of fatigue makes it difficult to assess and treat. Patients may have varying perceptions and experiences of fatigue, which can be influenced by cultural, social, and personal factors. Overall, the lack of a standardized definition, assessment tools, and treatment approaches for fatigue represents a significant challenge for clinicians and researchers working in this area. Nevertheless, efforts are underway to develop more robust and reliable methods for measuring and treating fatigue, with the goal of improving the quality of life and overall health outcomes for patients affected by this debilitating symptom.

In this study, the mean of the total MFI-K score of patients with IBD was high (59.0 ± 5.5). Although comparison was hampered by the lack of a control group, a study on the reliability and validity of the Korean version of the MFI reported scores of 52.85 ± 9.63 for moderate fatigue and 61.23 ± 10.54 for very severe fatigue [29]. Our finding of a high level of fatigue in IBD patients is in line with previous reports of a higher prevalence of fatigue in patients with IBD compared to the general population [2,3,12,33].

The reason for higher fatigue level in patients with IBD are multifactorial and not fully understood. One possible explanation is that IBD is associated with systemic inflammation, which can cause fatigue. Inflammatory cytokines, which are released in response to inflammation, have been linked to fatigue in other chronic illness populations, such as cancer patients and those with autoimmune disorders [34,35]. It is possible that the chronic inflammation in IBD contributes to the high levels of fatigue reported by patients. A study found that IBD patients with fatigue had higher levels of memory T cells and neutrophils but lower levels of monocytes, compared to those without fatigue [36]. Inflammatory cytokines such as tumor necrosis factor alpha (TNF-α), interferon gamma (IFNγ), interleukin (IL)-12, and IL-10 were also significantly higher in the fatigue group, while IL-6 was lower. Longitudinal data from another study also showed a strong association between fatigue and inflammation [37]. In a study of children with IBD, those with lower insulin-like growth factor I (IGF-1) z scores had greater fatigue and higher levels of IL-10, IL-17A, IL-6, and IFNγ, suggesting a role of inflammatory pathways in fatigue pathogenesis [38].

It is also worth noting that the relationship between fatigue and IBD is likely bidirectional. That is, not only can IBD cause fatigue, but fatigue can also exacerbate the symptoms of IBD. For example, fatigue can lead to decreased physical activity, which can worsen intestinal inflammation and contribute to disease flares [39].

We found that moderate-to-severe disease activity was independently associated with increased general and physical fatigue, while the use of biologics was associated with decreased fatigue. These results suggest that fatigue may increase with more severe disease activity and decrease when the disease is well controlled with biologics. The association between disease activity and fatigue is controversial. In a multicenter prospective study conducted in Spain, although depression, anxiety, lack of sleep, and extraintestinal manifestations were related to fatigue, disease activity and anti-TNF therapy were not related to fatigue, contrary to our results [23]. However, inflammation is a contributor to fatigue in patients with active disease [12]. Indeed, proinflammatory cytokines, including interleukin (IL)-1, IL-6, IL-12, and TNF, have been suggested as triggers of fatigue [36,40]. In addition, disease activity was associated with fatigue in multiple clinical studies of IBD patients with active disease [37,41,42]. Prior reports that fatigue decreased after the administration of infliximab are consistent with our findings [7,43]. Interestingly, the use of biologics was also associated with decreased mental fatigue. The mental fatigue subscale of the MFI-K assesses cognitive aspects of fatigue, such as difficulty in concentrating, problems with memory and thinking, and a feeling of mental exhaustion [29]. The results of our study suggest that controlling diseases with biologics may also help reduce mental fatigue.

Known risk factors for fatigue, such as anemia and hypoalbuminemia, did not significantly increase fatigue among the patients with IBD in this study. Although there is no clear explanation for this result, it is possible that anemia and hypoalbuminemia progress slowly in IBD patients, allowing them to adapt to low levels of hemoglobin and albumin and, thus, to develop tolerance to their fatigue. Indeed, anemia was not associated with fatigue in previous studies, in agreement with our finding [44,45,46]. In addition, there was a previous report that iron deficiency was not associated with fatigue levels [9]. However, in our patients, there may have been insufficient statistical power to detect a significant relationship because of the mildness of the anemia and hypoalbuminemia.

We identified that a history of surgery for IBD was significantly associated with a higher level of fatigue. While surgery is known to significantly impact the quality of life of patients with IBD, there has been little reporting on its association with fatigue. A study of 311 patients with IBD showed that IBD-related surgery was related to fatigue [47]. Surgery is a major stressor for the body and can lead to a systemic inflammatory response, which can contribute to fatigue. Additionally, surgery may cause physical deconditioning and a decrease in physical activity, which can exacerbate fatigue. Patients who have undergone surgery for IBD may also experience postoperative complications, such as infection, bleeding, or ileus, which can further contribute to fatigue. Furthermore, surgery for IBD often involves the removal of a portion of the intestine, which can affect nutrient absorption and result in malnutrition, leading to fatigue. Finally, the psychological impact of undergoing surgery and the uncertainty of the disease course may also contribute to fatigue.

In order to examine the impact of psychosocial factors that are known to be important contributing factors to fatigue, we measured anxiety and depression levels using HADS. Our analysis showed that depression was associated with a decrease in reduced motivation among the four subclasses of MFI-K. Previous studies consistently reported a significant positive correlation between fatigue and depression in patients with IBD [32,48], although the direction of causation remained unclear. Patients experiencing fatigue may be more prone to developing depression due to the negative impact of fatigue on the quality of life, daily functioning, and social relationships. On the other hand, depression can also contribute to the development of fatigue due to its association with various physical symptoms, such as sleep disturbances and appetite changes. While the exact nature of the relationship between fatigue and depression in IBD patients is not well understood, it is thought to be mediated by a range of biological, psychological, and social factors. Chronic inflammation, for instance, may play a role in both fatigue and depression in IBD patients, as it can lead to increased levels of fatigue and contribute to the development of depression [49]. Comprehensive treatment that includes medication, psychological support, and lifestyle changes may be necessary to address both fatigue and depression in patients with IBD.

This study had several limitations. First, the sample was small; further studies with more patients are needed to assess the degree of, and the risk factors for, fatigue in patients with IBD. Second, few of the subjects had severe IBD. The relationship between disease activity and fatigue could be more clearly delineated by further investigations that include patients with more severe IBD. Third, although our study conducted multiple statistical tests, we did not adjust for the significance level, considering the exploratory nature of the research and the limited sample size. We believed it was crucial to identify as many potential factors as possible to generate hypotheses for future studies. Fourth, although previous studies have suggested that sleep disorders may be a possible factor contributing to fatigue in patients with IBD [20,22,37], we were unable to investigate this possible factor in our study.

## 5. Conclusions

Fatigue is a common and debilitating symptom in patients with IBD, with significant negative consequences on the quality of life. Despite its importance, fatigue is often overlooked and difficult to treat. Although the cause of fatigue in patients with IBD is not fully understood, our study aimed to investigate potential contributing factors. Our results showed that moderate-to-severe disease activity was independently associated with increased general and physical fatigue, while the use of biologics was associated with decreased fatigue. Additionally, a history of surgery for IBD was found to be an associated factor for fatigue. Depression was mainly related to reduced motivation in the different subscores of fatigue. Our study is significant in that we explored factors associated with fatigue that could be potential therapeutic targets in the future. Further longitudinal studies are needed to investigate the causal relationship between the identified factors and fatigue.

## Figures and Tables

**Table 1:** Baseline characteristics of the study population.

	All IBD Cases (*n* = 147)	UC (*n* = 97)	CD (*n* = 50)	*p*-Value
Age (years)	36.2 ± 13.5	40.3 ± 13.8	28.7 ± 8.9	<0.001
Sex				0.098
Male	97 (66.0)	59 (60.8)	38 (76.0)	
Female	50 (34.0)	38 (39.2)	12 (24.0)	
BMI (kg/m^2^)	22.6 ± 3.7	22.7 ± 3.3	22.3 ± 4.5	0.587
Duration of disease (years)				
<1	42 (28.6)	25 (25.8)	17 (34.0)	0.471
1–5	59 (40.1)	42 (43.3)	17 (34.0)	
>5	46 (31.3)	30 (30.9)	16 (32.0)	
History of surgery for IBD	11 (7.5)	0 (0)	11 (22)	<0.001
Hemoglobin (g/dL)	13.8 ± 1.8	14.0 ± 1.7	13.5 ± 1.9	0.131
Protein (g/dL)	7.4 ± 0.6	7.4 ± 0.6	7.4 ± 0.7	0.703
Albumin (g/dL)	4.5 ± 0.5	4.5 ± 0.5	4.5 ± 0.5	0.412
ESR (mm/h)	13.0 ± 16.7	12.4 ± 14.3	14.4 ± 20.8	0.521
CRP (mg/dL)	4.8 ± 17.3	5.7 ± 20.1	3.2 ± 10.0	0.410
25-OH-Vitamin D (ng/mL)	29.4 ± 18.4	29.8 ± 17.8	28.7 ± 19.6	0.778
Current medication				
5-ASA	115 (78.2%)	82 (84.5%)	33 (66.0%)	0.018
Immunomodulator	65 (44.2%)	23 (23.7%)	42 (84.0%)	<0.001
Steroid	31 (21.1%)	14 (14.4%)	17 (34.0%)	0.011
Biologics	35 (23.8%)	16 (16.5%)	19 (38.0%)	0.007
Disease activity				
Remission	60 (40.8%)	32 (33.0%)	28 (56.0%)	0.001
Mild	41 (27.9%)	37 (38.1%)	4 (8.0%)	
Moderate	41 (27.9%)	24 (24.7%)	17 (34.0%)	
Severe	5 (3.4%)	4 (4.1%)	1 (2.0%)	
MFI-K	59.0 ± 5.5	58.8 ± 5.8	59.3 ± 4.9	0.605
HADS-A	6.5 ± 4.3	6.3 ± 4.1	6.9 ± 4.7	0.410
HADS-D	7.3 ± 4.3	6.9 ± 4.0	8.0 ± 4.9	0.124

Data were means ± SD or *n* (%). *p*-values were obtained by independent *t*-test or chi-squared test. IBD, inflammatory bowel disease; UC, ulcerative colitis; CD, Crohn’s disease; BMI, body mass index; ESR, erythrocyte sedimentation rate; CRP, C-reactive protein; 5-ASA, 5-aminosalicylic acid; MFI-K, Korean version of the Multidimensional Fatigue Inventory; HADS-A, Hospital Anxiety and Depression scale (HADS) anxiety subscale; HADS-D, HADS depression subscale.

**Table 2:** Total MFI-K scores and MFI-K subscale scores according to demographic characteristics.

			MFI-K Subscale							
	Total MFI-K	*p*-Value	General and Physical Fatigue	*p*-Value	Mental Fatigue	*p*-Value	Reduced Activity	*p*-Value	Reduced Motivation	*p*-Value
Age (years)		0.439		0.572		0.819		0.185		0.092
<40	58.7 ± 5.1		16.9 ± 2.0		17.6 ± 2.6		14.0 ± 2.1		10.3 ± 3.2	
≥40	59.5 ± 6.2		17.1 ± 1.9		17.5 ± 3.2		13.4 ± 2.3		11.4 ± 4.3	
Sex		0.473		0.614		0.209		0.029		0.140
Male	59.2 ± 5.8		17.1 ± 2.1		17.8 ± 3.0		14.1 ± 2.2		10.3 ± 3.7	
Female	58.5 ± 4.9		16.9 ± 1.7		17.1 ± 2.4		13.2 ± 2.2		11.3 ± 3.5	
BMI (kg/m^2^)		0.600		0.533		0.716		0.029		0.043
<25	59.3 ± 5.6		17.1 ± 1.9		17.6 ± 2.7		13.7 ± 2.2		11.0 ± 3.6	
≥25	58.7 ± 4.4		16.9 ± 2.1		17.8 ± 3.0		14.7 ± 1.9		9.4 ± 3.5	
Duration of disease (years)		0.788		0.065		0.980		0.980		0.876
<1	59.3 ± 4.5		17.5 ± 1.9		17.6 ± 2.6		13.8 ± 2.2		10.4 ± 3.5	
1–5	59.1 ± 5.8		17.1 ± 2.0		17.6 ± 3.1		13.8 ± 2.4		10.7 ± 3.5	
>5	58.5 ± 6.0		16.5 ± 1.9		17.5 ± 2.6		13.7 ± 1.9		10.8 ± 3.9	
History of surgery for IBD		0.085		0.767		0.654		0.539		0.084
No	58.8 ± 5.5		17.0 ± 1.9		17.5 ± 2.8		13.8 ± 2.2		10.5 ± 3.6	
Yes	61.7 ± 5.0		17.2 ± 2.4		17.9 ± 2.5		14.2 ± 1.8		12.5 ± 3.9	
Anemia		0.987		0.170		0.128		0.371		0.338
No	59.0 ± 5.4		16.9 ± 2.0		17.7 ± 2.8		13.8 ± 2.1		10.6 ± 3.6	
Yes	59.0 ± 5.9		17.5 ± 1.6		16.8 ± 2.8		13.4 ± 2.6		11.3 ± 3.8	
Hypoalbuminemia		0.896		<0.001		0.483		0.158		0.367
No	59.0 ± 5.5		16.9 ± 2.0		17.6 ± 2.6		13.8 ± 2.1		10.6 ± 3.6	
Yes	58.7 ± 7.7		18.3 ± 0.5		15.8 ± 5.7		12.5 ± 3.0		12.0 ± 5.1	
Steroid		0.299		0.376		0.607		0.677		0.657
No	58.8 ± 5.6		16.9 ± 2.0		17.5 ± 2.7		13.8 ± 2.1		10.6 ± 3.7	
Yes	59.9 ± 5.1		17.3 ± 1.9		17.8 ± 3.2		13.9 ± 2.5		10.9 ± 3.3	
Biologics		0.031		0.102		0.071		0.503		0.571
No	59.5 ± 5.5		17.2 ± 2.0		17.8 ± 2.8		13.9 ± 2.1		10.7 ± 3.7	
Yes	57.3 ± 5.0		16.5 ± 1.8		16.8 ± 2.7		13.6 ± 2.5		10.3 ± 3.4	
Disease activity		0.083		0.009		0.611		0.981		0.102
Remission and mild	58.5 ± 5.4		16.7 ± 2.0		17.6 ± 2.6		13.8 ± 2.1		10.3 ± 3.6	
Moderate and severe	60.2 ± 5.5		17.6 ± 1.7		17.4 ± 3.2		13.8 ± 2.3		11.4 ± 3.7	
Anxiety *		0.315		0.138		<0.001		<0.001		<0.001
No	59.4 ± 5.5		16.8 ± 2.1		18.4 ± 2.7		14.5 ± 1.9		9.7 ± 3.1	
Yes	58.4 ± 5.4		17.3 ± 1.6		16.2 ± 2.5		12.7 ± 2.3		12.2 ± 3.9	
Depression ^†^		0.846		0.122		<0.001		<0.001		<0.001
No	58.9 ± 5.5		16.8 ± 2.1		18.3 ± 2.6		14.4 ± 2.0		9.4 ± 3.3	
Yes	59.1 ± 5.5		17.3 ± 1.8		16.5 ± 2.7		13.0 ± 2.2		12.3 ± 3.4	

Data were means ± SD. *p*-values were obtained by independent *t*-test or one-way ANOVA. * The presence of anxiety was defined as a HADS-A score of 8 or higher. ^†^ The presence of anxiety was defined as a HADS-D score of 8 or higher. MFI-K, Korean version of the Multidimensional Fatigue Inventory; BMI, body mass index; IBD, inflammatory bowel disease.

**Table 3:** Multiple linear regression between the MFI-K scores and other variables of interest in IBD patients: total MFI-K score; general and physical fatigue score; mental fatigue score; reduced activity score; reduced motivation score.

	Total MFI-K		MFI-K Subscale							
			General and Physical Fatigue		Mental Fatigue		Reduced Activity		Reduced Motivation	
	Coefficient (95% CI)	*p*-Value	Coefficient (95% CI)	*p*-Value	Coefficient (95% CI)	*p*-Value	Coefficient (95% CI)	*p*-Value	Coefficient (95% CI)	*p*-Value
Age										
Sex (male vs. female)					−0.89 (−1.82 to 0.04)	0.061	−0.99 (−1.73 to −0.24)	0.010	1.14 (−0.11 to 2.38)	0.073
BMI									−0.12 (−0.28 to 0.03)	0.119
Duration of disease										
History of surgery for IBD	3.35 (0.01–6.70)	0.049							0.68 (−0.40 to 3.77)	0.113
Anemia										
Hypoalbuminemia										
Steroid										
Biologics	−2.68 (−4.81 to −0.54)	0.014			−1.22 (−2.21 to −0.23)	0.016				
Disease activity (moderate to severe vs. remission to mild)			0.86 (0.15–1.58)	0.019						
Anxiety *					−1.31 (−2.34 to −0.29)	0.012	−1.08 (−1.89 to −0.26)	0.010		
Depression ^†^			0.49 (−0.18 to 1.15)	0.152	−1.34 (−2.35 to −0.33)	0.009	−0.72 (−1.52 to 0.08)	0.077	2.86 (1.71–4.01)	<0.001

Note. Only variables with a *p* value less than 0.2 on simple linear regression were included in multiple regression analysis. * The presence of anxiety was defined as a HADS-A score of 8 or higher. ^†^ The presence of anxiety was defined as a HADS-D score of 8 or higher. MFI-K, Korean version of the Multidimensional Fatigue Inventory; CI, confidence interval; BMI, body mass index; IBD, inflammatory bowel disease.

## Data Availability

Data sharing was not applicable to this article.
